# Chitin Deacetylases: Properties and Applications

**DOI:** 10.3390/md8010024

**Published:** 2010-01-14

**Authors:** Yong Zhao, Ro-Dong Park, Riccardo A. A. Muzzarelli

**Affiliations:** Department of Agriculture Chemistry, Institute of Agricultural Science and Technology, Chonnam National University, Gwangju 500-757, Korea; E-Mail: zy2198@gmail.com (Y.Z.)

**Keywords:** chitin deacetylase, chitosan, chitooligosaccharides, degree of acetylation (DA), pattern of acetylation (PA)

## Abstract

Chitin deacetylases, occurring in marine bacteria, several fungi and a few insects, catalyze the deacetylation of chitin, a structural biopolymer found in countless forms of marine life, fungal cell and spore walls as well as insect cuticle and peritrophic matrices. The deacetylases recognize a sequence of four GlcNAc units in the substrate, one of which undergoes deacetylation: the resulting chitosan has a more regular deacetylation pattern than a chitosan treated with hot NaOH. Nevertheless plain chitin is a poor substrate, but glycolated, reprecipitated or depolymerized chitins are good ones. The marine *Vibrio* sp. colonize the chitin particles and decompose the chitin thanks to the concerted action of chitinases and deacetylases, otherwise they could not tolerate chitosan, a recognized antibacterial biopolymer. In fact, chitosan is used to prevent infections in fishes and crustaceans. Considering that chitin deacetylases play very important roles in the biological attack and defense systems, they may find applications for the biological control of fungal plant pathogens or insect pests in agriculture and for the biocontrol of opportunistic fungal human pathogens.

## 1. Introduction

The enzyme chitin deacetylase (EC 3.5.1.41) hydrolyzes the acetamido group in the *N*-acetylglucosamine units of chitin and chitosan, thus generating glucosamine units and acetic acid. It is one of the members of the carbohydrate esterase family 4 (CE-4s), as defined in the CAZY database (http://afmb.cnrs-mrs.fr/~cazy/CAZY) [1]. Members of this family share a conserved region in their primary structure, which has been assigned as the “NodB homology domain” or “polysaccharide deacetylase domain”. Besides chitin deacetylase, there are several other members in this family, including NodB protein (EC 3.5.1.-) [2] and peptidoglycan deacetylase (EC 3.1.1.-) [3,4].

Chitin deacetylase was first discovered from extracts of the fungus *Mucor rouxii* [5] and it was further found that the enzyme was associated with cell wall synthesis by converting nascent chitin into chitosan [6]. Since then, several different fungal chitin deacetylases have been identified, purified and characterized (Table 1).

Among them, *Colletotrichum lindemuthianum* chitin deacetylase has been the most well studied, including its biochemical properties [12,22], catalytic mechanism [23,24] and biological roles [25]. Through a series of kinetic studies, it was found that the deacetylation pattern of chitin deacetylase from *C. lindemuthianum* was totally different with that from *M. rouxii* (see below). Thus, based on the understandings on these functionally different enzymes, not only the degree of acetylation (DA) but also the pattern of acetylation (PA) of the enzymatic deacetylation products could be controlled during the enzymatic conversion from chitin or chitin oligomers to chitosan or chitosan oligomers.

Fungal chitin deacetylases have been studied more amply than those from insects and from marine bacteria. Even though several chitin deacetylase genes have been identified in insects, and it was proposed that chitin deacetylase may be widely present in insects, especially in the peritrophic midgut matrix, the biochemical properties of these chitin deacetylases have not been determined yet [26,27]. In this review we describe the occurrences of chitin deacetylases in marine invertebrates, in marine and terrestrial fungi, in insects and marine bacteria, along with their biochemical properties, modes of action, biological roles and applications.

## 2. Occurrence of Chitin Deacetylases

The occurrence of chitosan in insects and fungi, and the role of chitin deacetylase on the modification of newly synthesized chitin before its crystallization, have been discussed by Ruiz-Herrera *et al*. [28], among others.

### 2.1. Fungal Deacetylases

Chitin deacetylases from several fungi have been reported [5,8–11,15–22,25,28–30]. Based on their diverse locations in fungi, chitin deacetylases have been divided into two subgroups. *M. rouxii* and *A. coerulea* chitin deacetylases are secreted into the periplasm, and are thus called intracellular chitin deacetylases [5,8], while *C. lindemuthianum* and *A. nidulans* chitin deacetylases are secreted into the culture medium, thus being called extracellular chitin deacetylases [15,25].

Chitin deacetylases are secreted during an exclusive period corresponding to their special biological roles. Chitin deacetylases from fungal species might be secreted during different periods. For instance, an extracellular chitin deacetylase from *C. lindemuthianum* was exclusively secreted during fungal hyphae penetration into plants to modify chitin that could be recognized by a plant resistance system [13]. In contrast, an intracellular chitin deacetylase from *M. rouxii* was produced during fungal cell wall formation [6]. They were expressed exclusively during sporulation of *S. cerevisiae* [31] and produced during vegetative growth of *C. neoformans* [21]. In a more recent report, a chitin deacetylase gene was found to be specifically expressed during fruiting body development in the basidiomycete *F. velutipes* [20].

Marine fungi were isolated from the surface of bryozoan colonies collected in the North Sea by Meens *et al*. [32]; minimal medium supplemented with 0.5% of finely ground powder of either chitin (DD 37.4%) or chitosan (DD 71.2%) was used. Of 32 tested strains, 28 were able to grow on chitin, whereas 22 grew well on chitosan plates. The large number of strains capable to grow on both polysaccharides is justified by their natural habitat. Biosynthesis of chitin deacetylase was induced by both chitin and chitosan, that are recognized and deacetylated. Acetate was liberated by *Metarrhizium* sp.*, Trichoderma* sp.*, Fusarium* sp.*, Cladosporium* sp.*, Phoma* sp., *Aspergillus* sp., and others. Acetate seemed to depress the progress of the deacetylation beyond a certain degree. It was concluded that the marine environment enables fungi to use chitin for their needs, *via* the perception of the soluble *N*-acetylglucosamine dimer liberated by chitinases, and the adhesion to chitin debris or to the carapace of crustaceans. Their capacity to synthesize deacetylases would indicate that in the marine environment the decomposition of chitin according to the so called “chitosan pathway” takes place frequently.

### 2.2. Insect Deacetylases

Chitin deacetylases have also been detected in insects, including *Anopheles gambiae* [27], *Apis mellifera* [27], *Drosophila melanogaster* [33,34], *Helicoverpa armigera* [35], *Mamestra configurata* [36], Tribolium castaneum [27] and *Trichoplusia ni* [26]. Most of the reported insect chitin deacetylases are associated with the midgut peritrophic membrane (PM) and evenly distributed throughout the entire length of PM, as shown for the chitin deacetylases from *T. ni* [26], *H. armigera* [35], and *M. configurata* [36]. In addition, the presence of these enzymes in the midgut tissue of larvae was detected only during the feeding period. When the larvae had stopped feeding in their later stages, this protein became absent in the midgut tissue [26]. Although the involvement of chitin deacetylase in PM is viewed as a common feature of insects, the occurrence of chitin deacetylases in insect is not restricted to PM. For instance, in *D. melanogaster*, two chitin deacetylase-like proteins, CDA1 and CDA2 (serpentine and vermiform) were found to be associated with the tracheal extracellular matrix [33,34].

### 2.3. Deacetylases from Marine Bacteria

Vibrionaceae are marine bacteria widely distributed in all oceanic and estuarine waters, mainly responsible for recycling nitrogen present in chitinous debris slowly sedimenting in the water column (Ferguson *et al*.) [37]: their ecological and evolutionary aspects have been recently reviewed by Hunt *et al*. who proposed a chitin degradation pathway based on the comparison of 19 *Vibrio* and *Photobacterium* genomes with a detailed metabolic map assembled for *V. cholerae* from published biochemical, genomic, and transcriptomic results [38]. Further, to assess whether chitin degradation is a conserved property of Vibrionaceae, a set of 54 strains from 32 taxa were tested for the ability to grow on various forms of chitin. All strains grew on *N*-acetylglucosamine (GlcNAc); the majority of isolates grew on crab shell and squid pen chitins and contained chitinase A genes. Overall, chitin metabolism is a core function of Vibrionaceae. Interestingly, all strains grow on beta-chitin, but only a majority grows on alpha-chitin as well, due to better accessibility to the beta structure.

## 3. Properties of Chitin Deacetylases

Till now, chitin deacetylases have been isolated from several fungi, including *A. coerulea* [28], *A. nidulans* [15], *C. lindemuthianum* ATCC56676 [12], *C. lindemuthianum* DSM63144 [13], *F. velutipes* [20], *M. rouxii* [7], *R. circinans* [9], *R. nigricans* [10], *S. brevicaulis* [17] and *S. cerevisiae* [18]. The properties of these purified chitin deacetylases, such as molecular mass, optimum pH and temperature, effect of metal ions, and substrate specificity, have also been investigated. In addition, several chitin deacetylases were also characterized without a homogeneous purification, such as *M. anisopliae* [16] and *Mortierella* sp. DY-52 [11].

### 3.1. Molecular Mass

It can be clearly observed that the molecular mass for most of chitin deacetylases is in the range of 25–80 kDa, although a 150 kDa molecular mass was found in *C. lindemuthianum* DSM 63144 (Table 1). Most of the previously reported chitin deacetylases are glycoproteins and exist in the *N*-glycosylated form (20–70%). They often exist in multiple isoforms. For instance, five isoforms of chitin deacetylase were produced from the broad bean rust fungus of *Uromyces viciae-fabae*, ranging from 12.7 to 48.1 kDa [30]. In addition, three isoforms of chitin deacetylase from *M. racemosus* (64, 30 and 26 kDa) and four isoforms in *R. nigricans* (64, 35, 30 and 26 kDa) [39], three isoforms of chitin deacetylase (70, 37 and 26 kDa) in *M. anisopliae* [16] and two isoforms of chitin deacetylase (59 and 50 kDa) in *Mortierella* sp. DY-52 [11] have been reported. So far, the mechanism of these multiple isoforms of chitin deacetylase has not yet been well explained.

### 3.2. Optimal pH and Temperature

According to the reported results, the optimum pH of most extracellular chitin deacetylases is neutral or in the alkaline range from 7–12, while most intracellular chitin deacetylases have optimal pH values in the 4.5–6 range. The optimal temperature is 50–60 °C for nearly all enzymes (Table 1).

### 3.3. Enzymatic Deacetylation of Chitin

For enzymatic assay, glycol chitin, chitin-50 (DA 50%) and chitin oligomers (DP ≥ 2) were normally selected as standard substrates because they are soluble and armorphous and are easily deacetylated by all chitin deacetylases. Besides these substrates, fungal chitin and chitosan (DA 30%) could also be well deacetylated. Cai *et al*. reported that chitin isolated from the fungus *Aspergillus niger*, was a good substrate for chitin deacetylase [17]. The chitin deacetylase activity from the basidiomycete *F. velutipes* was determined as around 13-fold higher on substrate of chitosan (DA 30%) than glycol chitin [20]. On the contrary, chitin deacetylase was not effective on natural insoluble crystalline chitin. In order to obtain a better accessibility to the acetyl groups for the deacetylation, chitin was treated through various physical and chemical methods such as heating, sonicating, grinding, derivatization and interaction with saccharides. However, none of these pretreatment could effectively modify the natural chitin although it was reported that a decrystallized chitin with a very small particle size called superfine chitin after treatment with 18% formic acid appeared to become a better substrate for fungal chitin deacetylase [40].

### 3.4. Substrate Specificity

Caufrier *et al*. tested acetyl xylan, peptidoglycan and soluble chitin as substrates for chitin deacetylase from *M. rouxii* and both a native and a truncated form of acetyl xylan esterase from *Streptomyces lividans*. All enzymes tested were determined to be active on acetyl xylan and soluble chitin while inactive on peptidoglycan, which means that not only chitin but also acetyl xylan could be handled by chitin deacetylase [41]. This may be explained as that both chitin deacetylase and acetyl xylan esterase have a similar catalytic domain which is different from that of peptidoglycan deacetylase. Sequence alignment together with recently reported structural analysis suggests that one disulfide bond, tethering the *N*-terminal and *C*-terminal ends, is conserved in *M. rouxii* chitin deacetylase, *C. lindemuthianum* chitin deacetylase and *S. lividans* acetyl xylan esterase while absent from the homoglogous bacterial *Streptococcus pneumoniae* peptidoglycan deacetylase and *Bacillus subtilis* peptidoglycan deacetylase [22,42,43].

### 3.5. Influence of Metal Ions on Chitin Deacetylase

Chitin deacetylase has been suggested to be a metalloenzyme and its catalytic ability can be highly influenced by divalent cations. In previous reports, it was found that the enzyme activity of chitin deacetylase could be enhanced in the presence of Zn^2+^ (1 mM), Ca^2+^ (1 mM) and Co^2+^ (1 mM) [11,12,20]. Moreover, *N*-deglycosylation of Cda2p from *S. cerevisiae* resulted in complete loss of enzyme activity which could be restored by addition of 1 mM Co^2+^, while other metal ions such as Mg^2+^ and Mn^2+^ were proven to have not such activity restaurative effects [18]. Similarly, addition of EDTA eliminated total chitin deacetylase activity of basidiomycete *F. velutipes*, while the enzyme activity could be completely resotored by a simultaneous addition of Co^2+^ (1 mM) [20]. However, another report seems to tell another story: in the presence of Co^2+^ tested as the chloride salt, both *M. rouxii* chitin deacetylase and *S. lividans* acetyl xylan esterase exhibited increased activity on all soluble chitinous substrates, but did not exhibit increased activity on xylan as a substrate [41]. It seems that the influence of divalent cations on catalysis to some extent depends on the substrate tested. Zn^2+^ was also proved to be an important metal ion for chitin deacetylase.

Recently, the structure and catalytic mechanism of *C. lindemuthianum* chitin deacetylase have been reported [22]. The presence of a tightly enzyme-bound zinc ion was confirmed by both inductively coupled plasma mass spectrometric (ICP-MS) and graphite furnace atomic absorption spectroscopy (GF-AAS) analyses in agreement with the stuructural observations. The zinc ion is so tightly bound to the enzyme active site that it does not equilibrate with solution, hence the lack of *C. lindemuthianum* chitin deacetylase inhibition by EDTA [22].

### 3.6. Catalytic Mechanism

**Figure 1:**
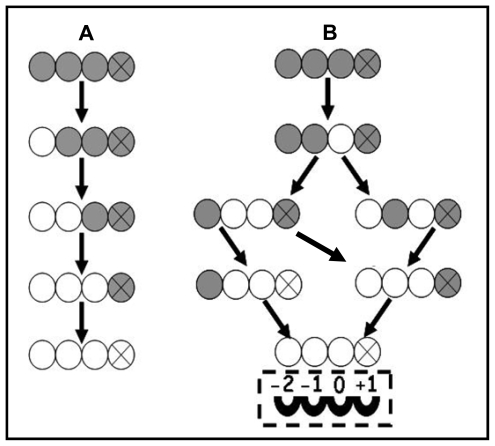
The pathway of (GlcNAc)_4_ deacetylation by an exo-type chitin deacetylase from *M. rouxii* (A) [45] and an endo-type chitin deacetylase from *C. lindemuthianum* (B) [23,24]. GlcNAc and GlcN are represented by shaded and open circles, respectively. The reducing end residue was indicated by the circle containing an X. The arrows indicated the sequence by which (GlcNAc)_4_ was deacetylated. In Figure 1A, (GlcNAc)_4_ was deacetylated by *M. rouxii* chitin deacetylase from the nonreducing end in a progressive multiple attack mode. In Figure 1B, (GlcNAc)_4_ was deacetylated by *C. lindemuthianum* chitin deacetylase in a multiple chain mode and four subsites are indicated as −2, −1, 0, +1: among them, only subsite 0 was responsible for the catalysis.

Chitin deacetylases from different sources show different enzymatic action patterns on chitin substrates. The mode of action of chitin deacetylase from *M. rouxii*, a Zygomycetes, has been studied on substrate of partially *N*-acetylated chitosans [44] and *N*-acetylchitooligosaccharides (DP 1–7) [45]. It was found that the exo-type enzyme hydrolyzed the acetyl groups of the substrates of either chitosan polymers or chitin oligomers according to a multiple attack mechanism in which binding of the enzyme on a chitin chain is followed by a number of sequential deacetylations after which the enzyme binds to another chain, as shown in Figure 1A.

The enzyme could only effectively deacetylate chitin oligomers with a DP higher than two and the first deacetylation takes place at the nonreducing-end residue of the oligomer. Among chitin oligomers (DP 1–7) tested, (GlcNAc)_4_ and (GlcNAc)_5_ could be fully deacetylated, whereas the reducing-end residue of (GlcNAc)_3_, (GlcNAc)_6_ and (GlcNAc)_7_ always remains intact [45].

Compared with *M. rouxii* chitin deacetylase, a more detailed investigation was carried out on *C. lindemuthianum* chitin deacetylase. In contrast to the results of *M. rouxii* chitin deacetylase, the extracellular chitin deacetylase from *C. lindemuthianum*, (ATCC 56676), a Deuteromycete, catalyzed the hydrolysis of acetamido groups according to a multiple chain mechanism. The enzyme could fully deacetylate (GlcNAc)_3_ and (GlcNAc)_4_ whereas the reducing-end residue (GlcNAc)_2_ could not be deacetylated [46]. In a further study, Tokuyasu and his colleagues carried out a structural analysis of the partially deacetylated products of (GlcNAc)_2–6_ formed by CDAH (the recombinant non-glycosylated chitin deacetylase from *C. lindemuthianum*). (GlcNAc)_4_ could be exclusively deacetylated to the product of GlcNAcGlcNAcGlcNGlcNAc by CDAH in an initial deacetylation process [23].

For a better understanding of the reaction mechanisms, it was proposed that the enzyme has four subsites (−2, −1, 0 and +1), as shown in Figure 1B. The enzyme strongly recognizes a sequence of four GlcNAc residues of the substrate, and the *N*-acetyl group in the GlcNAc residue positioned at subsite 0 is exclusively deacetylated. Among the four subsites, only subsite −2 strongly recognized the *N*-acetyl group of the GlcNAc residue of the substrate, while the deacetylation rate was not affected when either subsite −1 or +1 was occupied with a GlcN residue instead of GlcNAc residue [23]. Afterwards, to confirm the proposed subsites of the enzyme, a full steady-state kinetic characterization of CDAH was carried out [24]. The presence of four enzyme subsites that interact with GlcNAc residues from the non-reducing end to the reducing end of the substrate was experimentally confirmed. The turnover number is independent of n and represents the intrinsic rate constant for the hydrolysis of the acetamido group in subsite 0.

The steady-state kinetic parameters for the second deacetylation reaction of (GlcNAc)_4_ were also determined using (GlcNAcGlcNAcGlcNGlcNAc) as the substrate. The results suggest that the mono-deacetylated substrate binds strongly in a non-productive mode occupying all four subsites, thereby inhibiting the second deacetylation reaction [24].

In a more recent report, the structural data in combination with biochemical data reveal that the catalysis of *C. lindemuthianum* chitin deacetylase proceeds through a tetrahedral oxyanion intermediate. It can be proposed that the catalytic base Asp49 abstracts a proton from the water molecule, generating a nucleophile to attach the substrate carbonyl carbon. This produces a tetrahedral oxyanion intermediate, the charge of which is stabilized by the metal Zn and the backbone nitrogen of Tyr145. The pK_a_-tuned His206 then protonates the reaction intermediate on the nitrogen as it breaks down, generating a free amine and also the acetate product [22].

### 3.7. Gene and Structure

Chitin deacetylase genes have been cloned and characterized from several fungi and insects, including *C. lindemuthianum* [14,47], *D. melanogaster* [33,34], *F. velutipes* [20], *M. configurata* [36], *M. rouxii* [48], *R. circinans* [9], *R. nigricans* [10], *S. cerevisiae* [49], *S. pombe* [19], *T. castaneum* [27] and *T. ni* [26].

The enzymes are highly homologous and, furthermore, there is a universal conserved region that exhibits a significant similarity to the rhizobial nodulation proteins (NodB proteins), certain regions in microbial acetyl xylan esterases, xylanases, peptidoglycan deacetylase and several uncharacterized open reading frames (ORFs) in *Bacillus* sp. This conserved region has been assigned as the NodB homology domain [1,50].

Multiple sequence alignment of chitin deacetylase family demonstrates that the sequences contain five well conserved catalytic motifs which make up the active site of the deacetylase domain in the CE-4 family [22]. The five catalytic motifs cover several conserved histidine and aspartic acid residues, which were similarly observed in other CE-4 family members such as *S. pneumoniae* peptidoglycan deacetylase and *S. lividans* xylan esterase [42].

Motif 1 (TFDD) includes two aspartic acid residues; one interacts with zinc or cobalt and the second binds the acetate released from the substrate. Motif 2 (HSWSHP) contains two histidines that bind a metal ion and a serine or threonine that forms a hydrogen bond with the second histidine to stabilize the loop. Motif 3 (RPPY) forms one side of the active site groove and has multiple roles including binding acetate, binding zinc and coordinating the catalytic aspartate residue. The tyrosine residue was implicated in hydrogen bonding with acetate and the mutation of this residue to alanine inactivated the peptidoglycan deacetylase of *S. pneumoniae*. Motif 4 (DSLDW) forms the other side of the active site groove with the tryptophan being the most critical residue. Motif 5 (GSIVLMH) which includes a leucine and a histidine residue forms a hydrophobic pocket that binds the acetate methyl group and a histidine that forms a hydrogen bond with the product acetate [22,42].

It is worth mentioning that in *R. circinans*, three putative chitin deacetylase cDNAs have been isolated (RC, D2 and I3/2). However, after expression in the methylotrophic yeast *Pichia pastoris*, only RC chitin deacetylase was active. The inactive D2 and I3/2 chitin deacetylases were illustrated as a wrong choice of the secretion signal cleavage site, leading to an incorrect folding of the recombinant enzyme [9].

Guo *et al*. [26] were the first to isolate a cDNA encoding chitin deacetylase-like protein from a midgut cDNA expression library of the cabbage looper, *T. ni*. The chitin deacetylase-like protein, TnPM-P42, contains a putative polysaccharide deacetylase-like domain in the sequence from amino acid residues 54–186. However, chitin deacetylase activity was not detected. Luschnig *et al*. and Wang *et al*. characterized two *Drosophila* genes, *serpentine* (Dm*CDA1*, CG32209) and *vermiform* (Dm*CDA2*, CG8756), which encoded proteins with a chitin-binding domain (ChBD), a low-density lipoprotein receptor class A domain (LDLa) and a chitin deacetylase (CDA)-like catalytic domain. These researchers also reported a third gene encoding a CDA-like protein (ChLD3, Dm*CDA3*) that was expressed in epidermis during late stage embryogenesis [33,34]. This gene has not yet been characterized by mutational studies in detail.

In a recent report, a bioinformatics investigation of four insect species with annotated genome sequences identified a family of genes encoding CDA-like proteins, with five to nine members depending on the species [27]. These insect CDA-like proteins were further classified into five orthologous groups based on phylogenetic analysis. Group I and II contain, in addition to a polysaccharide deacetylase-like catalytic domain, a chitin-binding peritrophin-A domain (ChBD) and a low-density lipoprotein receptor class A domain (LDLa). Group III and IV also have ChBD but do not have LDLa domain. Group IV comprises CDA5s, which are the largest CDAs because of a very long intervening region separating the ChBD and the catalytic domains. Group V comprises CDA6–9 containing only a catalytic domain. Transcripts for the genes of this group are found in the larval gut but not in the carcass, suggesting that they are expressed predominantly in the gut [27]. Both fungal and insect chitin deacetylases have five motifs in the catalytic domain even though substitutions of several critical amino acids in these motifs often occur. In a more recent report, the functions of these chitin deacetylase gene family in *T. castaneum* were analyzed and the results indicated that specific ratios of chitosan-to-chitin may be critical for specific functions of particular chitin-containing body parts and for insect survival [51].

Blair *et al*. [22] described for the first time the crystal structure of a chitin deacetylase from *C. lindemuthianum* which was compared with that of two other members of the CE-4 family, *S. pneumoniae* peptidoglycan deacetylase [42] and *B. subtilis* peptidoglycan deacetylase [52]. All enzymes assume a tertiary structure consisting of a (β/a)_8_ fold, similar to the TIM barrel structure. In addition, the crystal structure of *C. lindemuthianum* chitin deacetylase reveals a well characterized zinc-binding motif (His-His-Asp) which is also conserved in other CE-4 family members with few exceptions [22,42]. Two charge relayed side chain pairs are observed, consisting of the catalytic base (Asp49) tethered by a conserved Arg142 and the catalytic acid (His206) tethered by a conserved Asp172, which perform acid/base catalysis using a water molecule as the nucleophile tightly associated to the zinc cofactor [22].

## 4. Biological Roles of Chitin Deacetylases

### 4.1. Biological Roles of Deacetylases from Marine Bacteria

Chitin is an abundant source of carbon, nitrogen, and energy for marine microorganisms, and *Vibrio cholerae* is a typical autochthonous member of diverse aquatic ecosystems around the globe. The interaction of *V. cholerae* with chitin is a paradigmatic bacterium-substrate interaction with complex and significant influence on the habits of the bacterium. As chitin being possibly the most abundant biopolymer in the aquatic environment, its association with *V. cholerae* has provided the bacterium with a number of advantages, including food availability, adaptation to environmental nutrient gradients, tolerance to stress and protection from predators. As reviewed by Pruzzo *et al*., interactions between *V. cholerae* and chitin occur at multiple hierarchical levels in the environment and include cell metabolic and physiological responses, e.g., chemotaxis, cell multiplication, induction of competence, biofilm formation, commensal and symbiotic relationship with higher organisms, cycling of nutrients, as well as pathogenicity for humans and aquatic animals [53].

Microarray expression profiling and mutational studies of *V. cholerae* growing on a chitin surface, or in the presence of the soluble chitin oligosaccharides (GlcNAc)_2–6_, GlcNAc, or the glucosamine dimer (GlcN)_2_ identified three sets of differentially regulated genes. Meibom *et al*. showed that the sensor ChiS regulates expression of the (GlcNAc)_2–6_ gene set, including a (GlcNAc)_2_ catabolic operon, two extracellular chitinases, a chitoporin; GlcNAc causes the coordinate expression of genes involved with chitin chemotaxis and adherence and with the transport and assimilation of GlcNAc; and (GlcN)_2_ induces genes required for the transport and catabolism of nonacetylated chitin residues [54].

One such gene, cod, encodes a chitin oligosaccharide deacetylase when cells are induced by chitobiose (GlcNH_2_)_2_, or crude crab shells. That deacetylase is secreted at all stages of growth by *V. cholerae*. It was cloned, overproduced, and purified to apparent homogeneity by Li X.B. *et al*.: it is virtually inactive with GlcNAc, and moderately active with colloidal chitin [55]. The deacetylase is very active with chitin oligosaccharides, that are converted to products lacking one acetyl group, because it hydrolyzes the *N*-acetyl group attached to the penultimate GlcNAc unit. The gene bank sequence data show that cod is highly conserved in Vibrios and Photobacteria. One such gene encodes a deacetylase isolated from *V. alginolyticus* that is specific for (GlcNAc)_2_, but inactive with higher oligosaccharides. The COD enzymatic products, GlcNAc-GlcNH_2_-(GlcNAc)_n_, closely resemble those obtained by hydrolysis of the chitooligosaccharides with Nod B: GlcNH_2_-(GlcNAc)_3–4_ that are key intermediates in the biosynthesis of Nod factors, critically important in communications between the symbiotic nitrogen fixing bacteria and plants. Conceivably, the oligomers generated by deacetylases play equally important roles in cellular communications.

For the enzymatic hydrolysis of chitin, early work established that at least two enzymes are required, a chitinase that mainly yields *N,N*′-diacetylchitobiose (GlcNAc)_2_, and a beta-*N*-acetylglucosaminidase that yields the final product GlcNAc. This pathway remained the central concept of the chitin catabolism through the 20^th^ century. A motif of complexity of the chitin catabolic cascade is the participation of deacetylases, as described in a review by Jung *et al*. that mentions the genes involved in the chitin catabolic cascade of Vibrios in an attempt to better understand the metabolic pathway of chitin [56].

In fact, the treatment of powdered chitin with crude *V. parahaemolyticus* solution yielded the heterodisaccharide beta-d-*N*-acetylglucosaminyl-(1,4)-d-glucosamine GlcNAc-GlcN, as the primary chitin degradation product. The extracellular enzymes involved in the production of this heterodisaccharide, a 92 kDa chitinase and a 46 kDa chitin oligosaccharide deacetylase, were isolated from the crude enzyme solution, and their hydrolytic specificities were elucidated. These studies by Kadokura *et al*. confirmed that the chitinase hydrolyzes chitin to produce (GlcNAc)_2_ whilst the deacetylase hydrolyzes the acetamido group at the reducing end unit of the latter [57]. In a subsequent study, Hirano *et al*. found that the heterodisaccharide is an inducer of the production of the two hydrolases, particularly chitinase [58]. Similar results for chitinase production were obtained with other chitin-decomposing *Vibrio* strains harboring the carbohydrate esterase family 4 COD gene; however, such an increase in chitinase production was not observed in chitinolytic *Vibrio* strains that did not harbor the COD gene. These results suggest that GlcNAc-GlcN is a unique inducer of chitinase production in *Vibrio* bacteria that have the COD-producing ability, and that the COD involved in the synthesis of this signal compound is one of the key enzymes in the chitin catabolic cascade of these bacteria.

*Vibrio vulnificus* is a Gram-negative marine bacterium that contaminates shellfish and causes highly lethal sepsis and destructive wound infections with severely rapid pathological progress. Lee *et al*. [59] evaluated *in vitro* and *in vivo* the activity against *V. vulnificus* of two water-soluble chitosans, namely the partially depolymerized 10 kDa chitosan and the 1 kDa hexamer both having degree of deacetylation ca. 90% in concentrations 0.5 to 10 mg/mL. Treatment with the 10 kDa chitosan resulted in significantly higher suppressive effects on the growth of *V. vulnificus* than treatment with the oligomer. The growth of *V. vulnificus* was inhibited within 1 h of treatment with the 10 kDa chitosan. Moreover, it inhibited *V. vulnificus*-induced cytotoxicity in human intestinal epithelial cells, while the oligomer did not. Furthermore, the administration of 10 kDa chitosan (0.1–0.5 mg per mouse) significantly increased the survival period of the infected mice. The number of viable *V. vulnificus* cells in the spleen, liver, small intestine, and blood was significantly lower in 10 kDa chitosan-treated mice than in untreated mice. Thus, partially depolymerized chitosan is a potential agent for the prevention and treatment of infection generated by *V. vulnificus*.

In a study by Chaiyakosa *et al*., chitosan was compared to chlorine for reducing infection by *V. parahaemolyticus* in the frozen shrimp factories [60]. Chitosan could kill *in vitro* more than 90% of *V. parahaemolyticus* cells, whereas chlorine completely eliminated this organism. In artificially inoculated shrimp, more than 90% reduction of *V. parahaemolyticus* was achieved by chitosan. A similar reduction was obtained by chlorine, however, at lower concentrations and less contact time. In naturally contaminated shrimp, neither agent completely eradicated *V. parahaemolyticus*, however, chitosan achieved a decrease of more than 60%. These results demonstrate the possibility of using chitosan to decontaminate pathogenic bacteria in the seafood factory, a change that would diminish health problems of the workers, considering that chlorine causes severe respiratory diseases.

### 4.2. Biological Roles of Fungal Deacetylases

In *M. rouxii* and *A. coerulea*, chitin deacetylase was localized near the periplasmic space in the mycelia and contributed to formation of chitosan in the cell wall from nascent chitin synthesized by chitin synthease [6,28]. Even though enzymology and cytology of chitin biosynthesis in fungi has been extensively studied, very little information exists on the correlation between chitin deacetylase and chitosan biosynthesis [7].

In order to understand the biological role of chitin deacetylase, chitosan was selected as the study target for investigation using the model yeast *S. cerevisiae*. In this yeast, chitin is an essential component for vegetative growth but chitosan is not. However, in spore wall formation, both chitin synthesis and chitin deacetylation are required. Chitin is synthesized by three chitin synthases Chs1, Chs2 and Chs3 in *S. cerevisiae*, among them Chs3 plays a major role. Conversion of chitin to chitosan by either Cda1 or Cda2 allowed to make the second layered structure of the spore wall next to the outer dityrosine layer. The chitosan based structure is important for spores to retain its structural rigidity and resistance to various stresses [31]. Although it has been speculated that the Cda1p protein deacetylates a specific substrate produced during sporulation and is important for the maturation of the spore wall outer layers, and Cda2p is responsible for the formation of the chitosan-containing spore wall layers, the role of two yeast chitin deacetylase Cda1 and Cda2 is still not clear [18].

It was reported that chitosan forms a layer of the ascospore cell wall in *S. cerevisiae* and is suggested to be in the bridges between individual spores. The results demonstrated that the interspore bridges can maintain a physical connnection between spores after they are released from the ascus [61]. In addition, a *cda1*^+^ encoded chitin deacetylase in a fission yeast *S. pombe* was identified and analyzed. It was found that spore formation of a *cda1*^+^ disruptant was abnormal and expression of cda1 mRNA increased during sporulation process, suggesting chitin deacetylase in *S. pombe* is required for proper spore formation [19].

In another report, four putative chitin deacetylases, named Cda1, Cda2, Cda3 and Fpd1, have been identified from *C. neoformans*, which is an opportunistic fungal pathogen that causes cryptococcal meningoencephalitis. The chitosan produced by enzymatic removal of acetyl groups from nascent chitin polymers has been implicated as an important component of the vegetative cell wall. Among the four chitin deacetylases, Cda1, Cda2 and Cda3 account for all of the chitosan produced during vegetatibe growth in culture, but the function for Fpd1 remains undetermined. Utilizing a collection of chitin deacetylase deletion strains, it was determined that during vegetative growth, chitosan helps to maintain cell integrity and aids in bud separation. Additionally, chitosan is necessary for maintaining normal capsule width and the lack of chitosan results in a “leaky melanin” phenotype [21].

Chitin deacetylase plays an important role in protecting pathogenic fungal hyphae from being lysed by secreted plant chitinases by transforming into chitosan the superficial chitin in the cell wall of plant pathogenic fungi, such as the wheat stem rust fungus *Puccinia graminis* f. sp. *tritici* and the broad bean rust fungus *U. fabae*, and the causative agents of anthracnose, *C. graminicola* and *C. lindemuthianm*. These fungal plant pathogens, when colonizing their host plant tissues, encounter an elaborate defence system consisting of chemically and physically performed resistance factors and of induced resistance reactions. Hydrolases, such as chitinases and β-1,3-glucanases, represent standard antifungal enzymes found in most plants. The endo-type chitinases from plants degrade fungal chitin into chitin oligomers which may further act as elicitors of active defense responses within the plant cells.

A successful fungal pathogen could evade plant antimicrobial hydrolases by enzymatic modification or affinity modulation. Based on the studies of cell wall composition of invasive fungal hyphae, it was suggested that exposed fungal chitin polymers are partially de-*N*-acetylated during the infection and initial growth within the host for escaping from plant antimicrobial hydrolases [62]. Based on the studies of catalytic mechanism of *C. lindemuthianm* chitin deacetylase, it was assumed that the partially deacetylated product from chitin by the chitin deacetylase should be a poor substrate for both chitinases and chitosanases [24]. One avirulence protein (Avr4), containing an invertebrate chitin-binding domain (CBM14), is believed to mask and protect fungal cell wall chitin against hydrolysis by plant chitinases accumulated during infection by affinity modulation [63].

### 4.3. Biological Roles of Insect Deacetylases

The roles of insect chitin deacetylases are not well understood. In *D. melanogaster*, two chitin deacetylase-like proteins, CDA1 and CDA2 (serpentine and vermiform), were found to be associated with the tracheal extracellular matrix and limited tube elongation, presumably by deacetylating the terminal *N*-acetyl-d-glucosamine that is extended to form the chitin chain [33,34]. Deacetylation increases the solubility and decreases the density of chitin fibrils *in vitro* and therefore may influence the structure and orientation of chitin fibrils in the cuticle. A chitin deacetylase, *Mc*CDA1, was identified from *M. configurata* PM [35]. This protein may be involved in altering the physical and chemical properties of the chitin in the PM by deacetylating *N*-acetyl-d-glucosamine. This would not only alter chitin fibril structure but also affect the binding of PM proteins, PM integrity and porosity. The expression of *McCDA1* and *TnPM-P42* [26] was restricted to the midgut. In addition, of the nine *T. castaneum* CDA genes, *TcCDA-6*, *-7*, *-8* and *-9* were exclusively expressed in the gut [27], suggesting that a subclass of chitin deacetylase play a role in gut physiology. Dixit *et al*. also speculated that Group V chitin deacetylase may be involved in insect immunity or may alleviate the inhibitory effect of chitooligosaccharides on the activity of gut chitinases needed for the moulting process [27].

A more recent proteomics analysis on PM proteins indicated that there were two major proteins, chitin deacetylase-like and mucin-like proteins, in chitin-containing structure of midgut and the former one may participate in immobilization of digestive enzymes, actively protect the gut from parasite invasion and intercept toxins such as lectins [35].

## 5. Applications of Chitin Deacetylases

Chitin deacetylases, based on their different catalytic mechanism and different biological roles, are potentially useful in several areas, as listed in Table 2.

### 5.1. Applications in the Marine Field

Multiple industrial and medical uses of chitin and its derivatives have been developed in recent years. The demand for enzymes with new or desirable properties continues to grow as additional uses of chitin, chitooligosaccharides, and chitosan become apparent. A review by Howard *et al*. summarized methods used to isolate and characterize chitin-modifying enzymes including chitinases, chitosanases, chitin deacetylases, *N*-acetylglucosaminidases, chitodextrinases, besides chitin-binding proteins [69].

It is important to note that *Vibrio* sp. recognize chitins and chitosans with opposite consequences: chitin supports their growth whilst chitosan depresses it. Therefore, the action of chitin deacetylases is finely tuned to the purposes of the biochemical needs of the *Vibrio* sp. It should be kept in mind that chitosans of various (high) deacetylation degrees as well as chitosans partially substituted with certain functional groups exhibit bactericidal/bacteriostatic action, as originally demonstrated by Muzzarelli *et al*. [70] and further confirmed by many laboratories around the world. Therefore an imbalance in the degree of acetylation would mean toxicity for the bacterium.

On the other hand, certain *Vibrio* strains produce internal or external infections in many marine organisms: for example, Vezzulli *et al*. investigated the role of surface membrane proteins in promoting attachment of various Vibrio strains to the copepod *Tigriopus fulvus* [71]. Sugita *et al*. clarified the abundance and taxonomic status of intestinal bacteria isolated from Japanese flounder *Paralichthys olivaceus*, and described their ability to digest chitin [72]. Phylogenetic analysis based on 16S ribosomal DNA sequences showed that 82 representative isolates were closely related to three major species of marine vibrios, *V. scophthalmi/V. ichthyoenteri* group (41 isolates), *V. fischeri* (39 isolates) and *V. harveyi* (two isolates), with similarities of 97.2–99.8%, 96.4–100% and 98.6–99.5%, respectively. These findings are similar to those for turbot, *Scophthalmus maximus*. Intestinal bacteria from several coastal fish species were screened by Itoi *et al*. on a medium containing 0.2% colloidal chitin: 361 bacteria capable of decomposing colloidal chitin were isolated [73]. They were then screened on media containing 0.5% of either alpha- or beta-chitin, resulting in the identification of 31 alpha-chitinolytic and 275 beta-chitinolytic bacterial isolates. Partial 16S rRNA gene sequencing was carried out: homology searches of the resultant sequences revealed that 99% of the chitinolytic bacteria isolated belonged to the Vibrionaceae.

In the light of this kind of data, Weinhold *et al*. recommended the use of well characterized chitosan for the purpose of studying growth inhibition of *Vibrio* strains. They analyzed the biological effects of chitosan, that revealed growth inhibition within 30 minutes for *E. coli* and a decreased bioluminescence for *V. fisheri* (IC_50_ = 0.035 w%) [74]. A number of laboratories are currently testing chitosan as an antibacterial agent to protect marine food: changes in microbial flora of Pacific oysters *Crassostrea gigas* during storage at 5 °C were analyzed by Cao *et al*. [66]: the dominant microorganisms were found to be *Pseudomonas* (22%) and Vibrionaceae (20%) in raw oysters. At the end of storage, *Pseudomonas* reached 73%, while Vibrionaceae remained at 20%. Wide-spectrum antibacterial property of chitosan against the bacteria isolated from oysters was observed, and chitosan concentration of 5.0 g/L was eventually determined for application in oyster preservation. Based on microbiological analysis, biochemical indices determination and sensory evaluation, the chitosan treatment prolonged the shelf-life of oysters from ca. 9 days to 15 days.

Nevertheless, chitin too can exert protective action. Adult male shore crabs *Carcinus maenas* were maintained by Powell *et al*. [67] on a fish-based diet supplemented with 5 or 10% chitin for 11 weeks. Significantly lower mortality was observed during this period in those fed 10% chitin compared to the control group (no chitin). Crabs fed 5 or 10% chitin had lower numbers of cultivatable bacteria in the hepatopancreas than those on the basal diet. The addition of chitin had no significant effect on the serum concentrations of protein and glucose, and the levels of glycogen in the hepatopancreas. The total number of circulating hemocytes in the blood was unaffected by the addition of chitin to the diet. The *in vitro* phagocytic activity of hemocytes was unaffected by chitin supplementation and crabs challenged with *V. alginolyticus* showed a similar pattern of susceptibility in the three dietary groups (0, 5 or 10% chitin). The enhanced survival of chitin-fed crabs probably results from the removal of pathogenic bacteria from the hepatopancreas consequent to the chitin recognition by *V. alginolyticus*.

### 5.2. Preparations of Chitosan from Chitin

The enzymatic deacetylation of various chitins was investigated by Aye *et al*. using the chitin deacetylase isolated from *Rhizopus oryzae* growth medium [64]. Chitin was confirmed to be a very poor substrate for the enzyme, but re-precipitated chitin was moderately better. Yamada *et al*. found that the recombinant *Flammulina velutipes* chitin deacetylase catalyses deacetylation of *N*-acetyl-chitooligomers, from dimer to pentamer, glycol chitin and colloidal chitin [75]. Chitosans with low DD can be further deacetylated with *Mucor rouxii* deacetylase as claimed by Martinou *et al*. [76,77].

In order to increase the efficiency of the enzymatic deacetylation for the production of chitosans, chitins were “modified” either physically or chemically by Beaney *et al*., and then they were reacted for 24 hr with extracellular deacetylase from *C. lindemuthianum* [78] (reportedly not inhibited by liberated acetate). Modifications of the chitins affected the degree of deacetylation to various extents: it was found that the dissolution and drying method used in modifying the chitins had significant impact on the final efficiency of the enzymatic deacetylation reaction. The most successful preparation made by freeze-drying a colloidal chitin suspension increased the degree of enzymatic deacetylation by 20 fold. The degree of crystallinity of the chitins must be reduced to enable enzymes to access the internal polysaccharide structure.

Spent *Aspergillus niger* mycelium from a citric acid production plant was used as a source of chitosan by Cai *et al*. The extraction of chitosan was operated with lysozyme, snailase, neutral protease and the chitin deacetylase from *Scopulariopsis brevicaulis* at the optimum condition of each enzyme. The optimum dosage of neutral protease and chitin deacetylase per 100 g of mycelium were 0.17 g (5100 units) and 1200 units, respectively. Molecular weight, degree of deacetylation of chitosan and the content of glucosamine were 268 kDa, 73.6% and 84.4%, respectively, but the deproteinization rate was deceptively at the 59.9% level [17]. Other authors too faced difficulties in removing proteins from chitosan with the aid of proteases. Therefore, a fully enzymatic process from raw material to chitosan is still unfeasible.

Jaworska *et al*. found that covalent binding of chitin deacetylase to a diethylaminoethyl cellulose *via* divinyl sulfone led to high activity and stability. The optimal pH was 4.0 for both enzymes and the optimal temperature 55 and 50 °C for free and immobilized forms, respectively. The kinetics of chitosan deacetylation for both enzymes followed the Michaelis-Menten equation, but significant differences in the values of the equation parameters were observed [65]. According to Li Z.L. *et al*., the best conditions for chitin deacetylase production from *Aspergillus nidulans* were the following: initial pH 6.5, carbon source 2%, nitrogen source 2%, the addition of metal ions 0.01 mol/L, inoculum 6%, and incubation at 31 °C for 96 hours [79].

Biotechnological approaches are more promising: for example, an expression plasmid containing the chitin oligosaccharide deacetylase gene from *V. parahaemolyticus* KN1699 was constructed by Kadokura *et al*. [80], and inserted into *E. coli* cells: the recombinant enzyme was secreted into the culture medium with the aid of the signal peptide. The concentration of the recombinant enzyme in the *E. coli* culture medium was 150 times larger than that of wild-type enzyme produced in the culture medium by *V. parahaemolyticus* KN1699. The recombinant enzyme was purified to homogeneity from culture supernatant in an overall yield of 16%. The wild-type and the recombinant enzymes had the same specificity.

Alfonso *et al*. found that acetate (0.4–4.0 mM) increased the hydrolytic capacity of chitin deacetylase isolated from *Aspergillus nidulans* when assayed on glycol chitin and chitin oligomers [15]. The *A. nidulans* chitin deacetylase was inactive on colloidal chitin and carboxymethyl chitin at lower rates, but it was inactive on GlcNAc, acrylamide, bisacrylamide, albumin and casein, thus confirming its high specificity. The optimum conditions were pH 7.0 and 50 °C; the chitin deacetylase was stable in the pH range 4–10 and the temperature interval 30–100 °C. These findings are remarkable in the light of the depression exerted by acetate on the enzymatic activity of the *M. rouxii* deacetylase, and the indifference of the *C. lindemuthianum* deacetylase to acetate. When deacetylating Antarctic krill *Euphausia superba* chitosans (DA 28 and 42%), the *M. rouxii* deacetylase activity was not appreciably inhibited by acetate, and both chitosans were deacetylated to DA 2 and 3% within 6 and 13 hr respectively, according to Martinou *et al*. [77,81]. It is supposed that origin and preliminary chemical deacetylation treatment of chitosans can make the kinetic data scarcely reproducible; in fact, the acetylation patterns of the krill chitosans (no longer commercially available) were later found to be far from uniform due to the presence of highly acetylated chain segments.

A major expectation in the practical use of chitin deacetylases was and still is to simplify the harsh chemical conversion process of chitin into chitosan, that moreover is not an environmentally friendly one. The enzymatic deacetylation would provide more regularly deacetylated chitosans, because as a point of difference from the chemical deacetylation, it does not proceed in a randomly (see above). As a second choice alternative, it would permit to deacetylate to a greater degree the partially deacetylated chitins coming from a mild chemical deacetylation process. Unfortunately it seems that some methodological error has been made in the past: for example nearly all reports deal with chitin/chitosan samples of the most various origins whose main characteristics are omitted, and in general only one sample was studied in each publication; no uniform protocol was adopted for the isolation of the deacetylases. It would have been wiser to study a number of well characterized chitins from various sources in each research project. As a consequence, contradictions can be found when the deacetylase inhibition is attributed to chitosan itself or to acetate, and when the performances are evaluated and compared for various deacetylases; even the MW values are questionable in most articles. The applications of chitin deacetylases in this area remain confined to the laboratory scale.

### 5.3. Applications in the Biochemical Area

In the biological control of fungal human pathogen, the fungal cell wall is an excellent target for antifungal therapies as it provides cell structure and integrity. It is needed for the localization or attachment of known virulence factors, including the polysaccharide capsule, melanin, and phospholipase, and it is critical for host-pathogen interactions. The chitosan produced by the enzymatic removal of acetyl groups from nascent chitin is an important component of the cell walls of certain fungi and helps maintain cell integrity. Thus, chitin deacetylases and the chitosan made by them may prove to be excellent antifungal targets [21,29,68].

The inhibition of chitin deacetylase could support the fungal cell wall hydrolysis by plant chitinases, thus the control of the plant pathogenic fungi becomes feasible [12]. Similarly, chitin deacetylase could also be a versatile tool in the biological control of insect pests. For instance, chitin deacetylase proved to be important in initiating pathogenesis of *M. anisopliae* (a kind of insect-pathogenic fungus) by softening the insect cuticle to aid mycelial penetration. Chitin deacetylase herein may have a dual role in modifying the insect cuticular chitin for easy penetration of fungal pathogen as well as in altering its own cell walls for defense from insect chitinase [16].

In the biological control of pest insects, chitin deacetylase proved to be a potential target for an insecticide. Chitin deacetylase is a major protein secreted in the peritrophic matrix of the arthropod gut during feeding; it can modify the chitin component in such a way as to protect the gut from parasite invasion, and intercept toxins like lectins. Thus, the inhibition of this enzyme represents a potential way to control the pest insects.

## Figures and Tables

**Table 1:** The biochemical properties of chitin deacetylases from fungi.

Fungi	Phylum	Optimal pH/Temp. (°C)	pI	Molecular Weight (kDa)	Carbohydrate contents (%)	Refs

*Mucor rouxii*	Mucoromycotina	4.5, 50	3.0	75–80	30	[7]
*Absidia coerulea*	Mucoromycotina	5.0, 50	NA	75	NA	[8]
*Rhizpus circinans*	Mucoromycotina	5.5–6.0, 37	NA	75	NA	[9]
*Rhizopus nigricans*	Mucoromycotina	NA	NA	100	53	[10]
*Mortierella sp. DY-52#*	Mucoromycotina	5.5, 60	NA	50, 59	NA	[11]
*Colletotrichum lindemuthianum (ATCC 56676)#*	Ascomycotina	12, 60	3.7	32–33	NA	[12]
*Colletotrichum lindemuthianum (DSM 63144)#*	Ascomycotina	8.5, 50	3.0	150	67	[13]
Colletotrichum lindemuthianum (UPS 9) #,*	Ascomycotina	8.0, 60	NA	25	0	[14]
*Aspergillus nidulans #*	Ascomycotina	7.0, 50	2.8	27	28	[15]
*Metarhizium anisopliae #*	Ascomycotina	8.5, NA	3.6	70, 37, 26	NA	[16]
*Scopulariopsis brevicaulis*	Ascomycotina	7.5, 55	NA	55	NA	[17]
*Saccharomyces cerevisiae Cda2p*	Ascomycotina	8.0, 50	NA	43	18	[18]
*Schizosaccharomyces pombe*	Ascomycotina	NA	NA	NA	NA	[19]
*Flammulina velutipes*	Basidiomycotina	7.0, 60	NA	31	0	[20]
*Cryptococcus neoformans*	Basidiomycotina	NA	NA	NA	NA	[21]

# , extracellular chitin deacetylases;

* , structure available;

NA, not available.

**Table 2:** Several potential applications of chitin deacetylases.

Applications	Refs.
Preparations of chitosan from chitin	[11,40,64,65]
Protection of fishes and crustaceans *via* inhibition of *Vibrio* deacetylases	[66,67]
Biological control of some pest insects	[16]
Applications as target for biological control of fungal human/plant pathogens	[12,21,29,68]
